# The significance of calcium-sensing receptor in sustaining photosynthesis and ameliorating stress responses in plants

**DOI:** 10.3389/fpls.2022.1019505

**Published:** 2022-10-11

**Authors:** Rui Bai, Chunming Bai, Xiaori Han, Yifei Liu, Jean Wan Hong Yong

**Affiliations:** ^1^ College of Land and Environment, National Engineering Research Center for Efficient Utilization of Soil and Fertilizer Resources, Northeast China Plant Nutrition and Fertilization Scientific Observation and Research Center for Ministry of Agriculture and Rural Affairs, Key Laboratory of Protected Horticulture of Education Ministry and Liaoning Province, Shenyang Agricultural University, Shenyang, China; ^2^ National Sorghum Improvement Center, Liaoning Academy of Agricultural Sciences, Shenyang, China; ^3^ The University of Western Australia (UWA) Institute of Agriculture, The University of Western Australia, Perth, WA, Australia; ^4^ School of Biological Sciences, The University of Western Australia, Perth, WA, Australia; ^5^ School of Agriculture and Environment, The University of Western Australia, Perth, WA, Australia; ^6^ Department of Biosystems and Technology, Swedish University of Agricultural Sciences, Alnarp, Sweden

**Keywords:** abiotic stress, photosynthesis, calcium, biological functionality, calcium-sensing receptor

## Abstract

Calcium ions (Ca^2+^) regulate plant growth and development during exposure to multiple biotic and abiotic stresses as the second signaling messenger in cells. The extracellular calcium-sensing receptor (CAS) is a specific protein spatially located on the thylakoid membrane. It regulates the intracellular Ca^2+^ responses by sensing changes in extracellular Ca^2+^ concentration, thereby affecting a series of downstream signal transduction processes and making plants more resilient to respond to stresses. Here, we summarized the discovery process, structure, and location of CAS in plants and the effects of Ca^2+^ and CAS on stomatal functionality, photosynthesis, and various environmental adaptations. Under changing environmental conditions and global climate, our study enhances the mechanistic understanding of calcium-sensing receptors in sustaining photosynthesis and mediating abiotic stress responses in plants. A better understanding of the fundamental mechanisms of Ca^2+^ and CAS in regulating stress responses in plants may provide novel mitigation strategies for improving crop yield in a world facing more extreme climate-changed linked weather events with multiple stresses during cultivation.

## Introduction

Calcium, a crucial macronutrient for plants, has important physiological functions such as maintaining cell morphology and regulating ion balance and osmotic pressure. Interestingly, it is also a key component for several early signaling pathways involved in plant–environmental stresses interactions (Lambers and Oliveira, 2019; Song et al., 2020; Wu et al., 2020). Most elemental calcium in plant cells exists in ionic forms, which contribute to forming microtubules in the cytoskeleton and maintaining the fluidity of cell membranes (Kong et al., 2020; Liu, 2021). In addition, calcium signals assist in the integration of various signaling pathways to coordinate plants’ growth response to multiple environmental signals (Edel and Kudla, 2016; Kudla et al., 2018; Luan and Wang, 2021; Kim et al., 2022). The extracellular calcium-sensing receptor (CAS) is an important protein that specifically exists in plants and has specific regulatory functions. These proteins regulate intracellular Ca^2+^ responses by sensing changes in extracellular Ca^2+^ concentration, thereby affecting a series of downstream signaling activities and fine-tuning plants’ responses to environmental perturbations. We summarized the research progress of Ca^2+^ and CAS in regulating photosynthesis and stress responses. Here, we focused on the fundamental role of CAS in regulating growth and signal transduction in response to the external environment.

## Structural characteristics of the calcium-sensing receptor

### Discovery and location of CAS

The extracellular Ca^2+^ receptor gene was cloned for the first time using the functional gene screening method in *Arabidopsis thaliana* and named as the calcium-sensing receptor (CAS) (Li et al., 2022). With the aid of fluorescence imaging and proteomics technologies, the CAS was located on the chloroplast thylakoid membrane of *Arabidopsis thaliana*, spinach, and green algae (Allmer et al., 2006; Naumann et al., 2007; Nomura et al., 2008; Weinl et al., 2008). Interestingly, the CAS was also detected within the purified eyespot of green algae (Trippens et al., 2017). Yamano et al. (2018) fused the CAS-Clover with high-resolution fluorescence imaging to coincide with the eyespot, which also verified the existence of CAS. In addition, high-resolution fluorescence images of CAS were obtained using the sensitive hybrid detector and image deconvolution technology, and its cellular localization on the thylakoid membrane was authenticated and observed with greater clarity.

### Structural features of CAS

The carboxyl-terminal of CAS is located within the cell and has two domains (Vainonen et al., 2008); one of which is a non-catalytic thiocyanate domain with high homology, and the other is related to protein interaction which can interact with the 14-3-3 and FHA (forkhead-associated) domain. Under light conditions, the CAS light-regulated kinase STN8 is targeted for phosphorylation at Thr-380 (Vainonen et al., 2008). The amino terminal of CAS is located outside the cell membrane, which may plausibly be the Ca^2+^ binding region, although its conserved sequence has not been found. It was later verified that the binding site of Ca^2+^ in CAS was the amino end rather than the carboxyl end in *Arabidopsis* and *Chlamydomonas* (Wang et al., 2016a).

## CAS and stomatal functionality

The pair of guard cells have a fundamental role in regulating gas exchange between plants and the atmosphere, and biotic infection (Melotto et al., 2008; Chen et al., 2012; Sawinski et al., 2013; Sun et al., 2014; Murata et al., 2015; Agurla et al., 2016; Bharath et al., 2021). Generally, Ca^2+^ plays an important role in regulating guard cell turgor and the movements of guard cells. Ca^2+^ ions affect stomatal conductance to water vapor and CO_2_ by regulating the pore size. Peng et al. (2022) discovered that an exogenous application of 18 Mm Ca^2+^ could increase the stomatal conductance of *Paris polyphylla* under high temperatures and strong light conditions. The CO_2_-induced stomatal closure process is also calcium-dependent and the CPK (calcium-dependent protein kinase) has a role in signal transduction and regulating the ion channels of guard cells. In the pentaploid mutant plants with *cpk3/5/6/11/23*, the stomatal movements were significantly impaired, suggesting that CPK has important functions in facilitating stomatal movement and is plausibly regulated by CO_2_ concentration (Schulze et al., 2021). Jakobson et al. (2016) confirmed that there were significant differences in stomatal phenotypes among *cas* mutants with different alleles and the *cas* overexpression of *Arabidopsis* transformants promoted stomatal closure. Li et al. (2017) also demonstrated that the deletion of the CAS gene could disrupt stomatal closure and this phenomenon was attributed to high levels of extracellular Ca^2+^. It was later verified that the extracellular high Ca^2+^ in CAS gene-deficient mutants could not induce stomatal closure, indicating that CAS was essential for extracellular Ca^2+^-induced stomatal closure in *Arabidopsis* (Qi et al., 2018).

**Figure 1:**
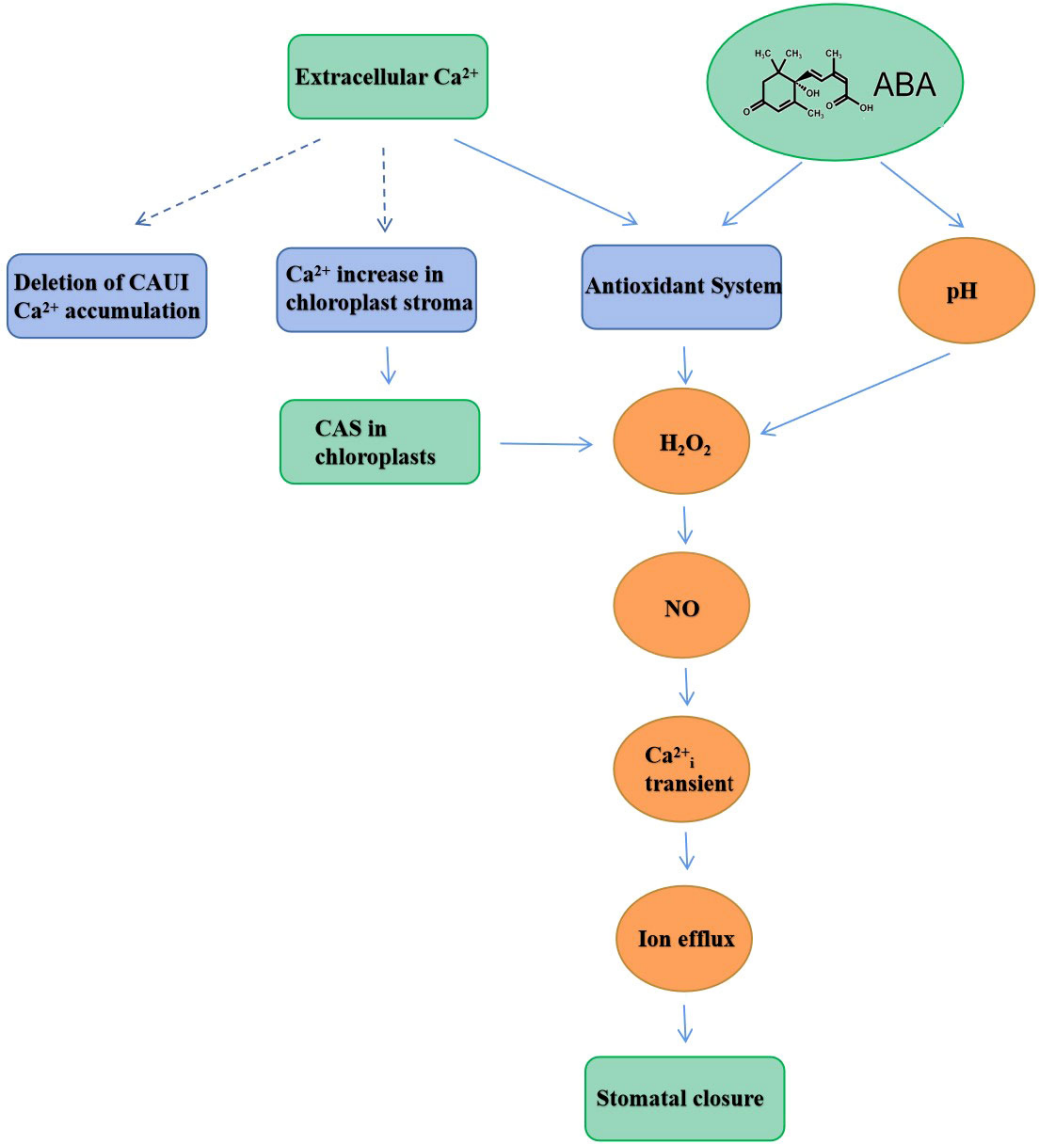
A simplified scheme illustrating the salient signal transduction steps leading to stomatal closure.

Interestingly, it was also found that CAS has an important role in elevating intracellular Ca^2+^ concentration when induced by extracellular Ca^2+^. These studies provided evidence for *Arabidopsis* chloroplasts in regulating extracellular Ca^2+^-induced intracellular Ca^2+^ concentration increases and subsequent stomatal closure (Nomura et al., 2008; Li et al., 2022). Wang et al. (2016b) found that the chloroplastic regulation of stomatal closure required the reduction of quinone, foliar production of H_2_O_2_, and the phosphorylation of CAS and LHCII during exposure to unfavorable perturbation. It was confirmed that stomatal movement was associated with NO and the NO acted downstream of the H_2_O_2_ step in regulating stomatal movement (Bright et al., 2006). Specifically, the stomatal closure induced by H_2_O_2_ and NO requires the involvement of extracellular Ca^2+^ and the accumulation of H_2_O_2_ and NO in guard cells (Li et al., 2009; Zhao et al., 2011). Tang et al. (2007) suggested that CAS might regulate the stomatal closure process through the Ca^2+^-CAS-IP signaling pathway. Subsequently, Wang et al. (2012) found that the extracellular Ca^2+^ induced H_2_O_2_ and NO accumulation through CAS in *Arabidopsis thaliana*, which induced transient changes in cytoplasmic Ca^2+^ concentration and was able to regulate stomatal movement. In addition, *Arabidopsis* plants expressing PH_PLCδ_-GFP protein were used for PH_PLCδ_-GFP imaging of guard cells, which indicated that H_2_O_2_ and NO were involved in the CAS-IP_3_ signaling pathway (Wang, 2013). Li et al. (2017) found that H_2_O_2_ might directly or indirectly regulate CAS activity and serve as redox signaling molecules, thereby regulating stomatal closure. Interestingly, the data also revealed that the exogenous NO-induced stomatal closure of CAS guard cells was facilitated by exogenous NO bypassing the damaged CAS. Furthermore, it was indicative that NO has a downstream role relative to CAS in the signal transduction pathway. Recent studies suggested that stomatal movement induced by extracellular Ca^2+^ was the culmination of multiple interactions among CAS, H_2_O_2,_ and NO signals (Zhang et al., 2018; Li et al., 2022). Li et al. (2017) used multi-disciplinary approaches to examine the responses of different mutants to extracellular Ca^2+^ in guard cells. It was found that there was a cascade of H_2_O_2_, ABA, and NO in the CAS signaling pathway. With further studies, it was confirmed that seed germination and stomatal closure of mutant cau1 transformants were excessively sensitive to ABA. In addition to extracellular Ca^2+^, the CAU1-CAS could also respond to ABA through interaction, and ABA and CAUI could also regulate stomatal closure (Ueda and Seki, 2020), although the specific role of CAS in this process required further clarification. Li et al. (2017) found that ABA in *CASas* mutant plants was able to induce stomatal closure, indicating that the ABA-induced signal transduction pathway was not dependent on CAS; here, *CASas* refers to the CAS antisense line of *Arabidopsis*. Based on these studies, the signal transduction cascade is summarized accordingly in a scheme (**Figure 1**).

## CAS and photosynthesis

Photosynthesis is one of the most important biological processes on earth (Liu et al., 2011; Stael et al., 2012; Lambers and Oliveira, 2019). Calcium is involved in regulating various photosynthetic processes, such as CO_2_ fixation and protein phosphorylation in chloroplasts (Rocha and Vothknecht, 2012; Liu, 2020). Hu et al. (2022) found that low calcium levels caused the decline in photosynthesis of *Taxus wallichiana* varieties, but this negative effect could be reversed by applying supplementary calcium nutrition, which was consistent with previous findings in grape and cucumber (He et al., 2018; Duan et al., 2020). Harnessing the pull-down analysis (network database GeneCAT) combined with proteomics and gene co-expression method, Wang (2013) found that 52% of the CAS co-expression genes in *Arabidopsis* were related to photosynthesis. Combining photosynthetic indexes, chloroplast structural observation and gene expression profiling, these observations verified that the photosynthetic level and biomass accumulation of *Arabidopsis CASas* plants were significantly reduced. Specifically, the inhibition of CAS caused the transcription level of photosynthetic electron-related genes to be down-regulated (Wang et al., 2014). Navazio et al. (2020) found that the transcription expression of the CAS gene in *Arabidopsis* was up-regulated under strong light conditions, and the CAS transcription level under dark and weak light conditions was also significantly up-regulated compared with that under normal light conditions. Petroutsos et al. (2011) confirmed that CAS played a role in maintaining PSII functionality and helping plants to adapt to strong light. Additionally, Li (2021) found three *AtCAS* homologous genes in some shade plants such as ginseng, and two *AtCAS* homologous genes in the non-shade plant such as tomato. As CAS is a phosphorylated protein regulated by light intensity; it is plausible that CAS may be closely related to light adaptability. The stomatal movement of terrestrial plants regulates CO_2_ entry and exit through HTI protein kinase and carbonic anhydrase (Hu et al., 2010), and aquatic plants maintained stable photosynthesis through the CO_2_-concentration mechanism (CCM). Studies have indicated that Ca^2+^ and CAS are related to CCM. In response to low CO_2_ concentration, the CAS can be transferred to the protein nucleus, and then mediate the chloroplastic retrograde signaling to maintain the expression of HLA3 (high-light activated 3) and LCIA (low-CO_2_-inducible gene A) (Wang et al., 2016a). Yamano et al. (2018) obtained interesting CAS-Clover high-resolution fluorescence images using a sensitive mixed detector coupled with image deconvolution technology. It was found that CAS moved along the thylakoid membrane in response to low concentration CO_2_, and gathered spatially in the protein nucleus during the CCM mechanism. As a chloroplastic thylakoid membrane protein, CAS functionality was examined during de-etiolation and chloroplast development in *Arabidopsis thaliana* (Huang et al., 2012). The thylakoid-localized CAS protein could assist in the production of cytosolic calcium transients, and the activation of the MPK3/MPK6 signal system. Subsequently, the activated MPK3/MPK6 was involved in the nucleus-induced phosphorylation of ABI4. The activation of ABI4 at both the transcriptional and posttranslational levels induced the inhibition of LHCB (Gao et al., 2016). These results showed that the cystoid-located CAS was involved in the retrograde signaling pathway from chloroplast to nucleus (Li et al., 2022).

Protein phosphorylation is vital for photosynthesis and adaptation to multiple environmental stresses (Vener, 2007; Fristedt et al., 2010). Vainonen et al. (2008) found that CAS could be phosphorylated by STN8, and the phosphorylation intensity increased with light intensity enhancement. At the same time, a new phosphorylation site Thr-367 was also found in the CAS of green algae (Lemeille et al., 2010). Cutolo et al. (2019) conducted proteomics analysis and phosphorylation determination of *CASas*, and identified Thr-376, Ser-378, and Thr-380 as the main phosphorylation sites of STN kinase. Protein phosphorylation is also important to regulate photosynthetic electron transport efficiency (Reiland et al., 2011). The cyclic electron transport (CET) process is involved in chloroplast energy regulation and redox metabolism, which is particularly important for photosynthesis. The studies demonstrated that CET has two pathways, PGR5/PGRL1-mediated pathway and the NDH-mediated pathway (Johnson et al., 2014; Ma et al., 2021a). The protein interaction between CAS and PGRL1 was confirmed in *Chlamydomonas reinhardtii*, indicating that there is a link between CAS and CET (Terashima et al., 2012; Ma et al., 2021b). Chen et al. (2015) found that Ca^2+^ controlled the biosynthesis of lipids induced by chloroplastic nitrogen starvation by promoting the expression of CAS in the PGRL1-mediated CET pathway.

## CAS, stress resilience, and plant defense

When plants are exposed to environmental stresses, they will improve their resilience by self-regulating several *in vivo* processes (e.g. changes in intracellular calcium concentration) to cope with these perturbations (Liu, 2020). Salinity is an important environmental factor, which has a negative impact on plant growth and photosynthesis (Acosta-Motos et al., 2017; Ma et al., 2022). Similarly, Ca^2+^ is also crucial in mediating stress responses (Park et al., 2016). It was verified that Ca^2+^ can enhance the salt tolerance of plants by improving water balance, regulating sodium secretion, and enhancing membrane integrity (Ahmed et al., 2021). In addition, Ca^2+^ also enhances resilience to chilling stress. Liu et al. (2013) found that exogenous Ca^2+^ could mitigate the significant decline in peanut photosynthesis and biomass accumulation during exposures to low night temperatures. It was also confirmed that exogenous Ca^2+^ ameliorated night chilling-dependent feedback inhibition of photosynthesis by improving sink demand and facilitating nonstructural carbohydrate export from chloroplasts, to restore peanut growth, dry matter production, and leaf photosynthetic capacity (Shi et al., 2020; Song et al., 2020; Wu et al., 2020). Sun et al. (2022) reported that the Ca^2+^ mediated-CAS was crucial for alleviating photoinhibition in peanut growing under conditions of moderate phosphorus deficiency. In addition, Zhao et al. (2015) confirmed that *OsCAS* could improve drought tolerance in *Arabidopsis*. When drought and heat stresses were imposed on the creeping bentgrass (*Agrostis stolonifera*), the expression of CAS was down-regulated, indicating that the calcium sensitivity to drought and heat stress responses was reduced (Xu and Huang, 2018). The molecular mechanism of proline metabolism regulation under drought also revealed a new drought tolerance pathway mediated by CAU1 (Fu et al., 2018). Huang et al. (2012) provided further evidence to support the role of Ca^2+^ and CAS in leaf re-greening and chloroplast development of *Arabidopsis*. Zheng et al. (2017) screened a chloroplast protein QUA1 by harnessing the large-scale forward genetic method. It is plausible that the CAS-mediated salt and drought tolerance of plants through the calcium signaling-linked QUA1 that influence the stability of CAS. It was further suggested that CAS has a regulatory role in the downstream of QUA1. Interestingly, there are some pieces of evidence to indicate that CAS is a prerequisite for chloroplast Ca^2+^ to induce light response and light-dark transition (Nomura et al., 2012). The expression of *cas* in *Arabidopsis thaliana* was significantly reduced under high-temperature treatment. It is plausible that the heat-sensing ability of chloroplasts was partially dependent on CAS during high-temperature exposure (Lenzoni and Knight, 2019).

Plant defense against invasive pathogens is dependent on the dual innate immune system. Meta-analysis indicated that CAS regulated the expression of flg22-induced immune genes through ^1^O_2_-mediated retrograde signaling; flg22 is a peptide derived from bacterial flagellins. These studies revealed that the weakened signals, chloroplasts, and superoxide anions were collectively involved in the innate immune system (Sano et al., 2014; Stael et al., 2015). In the study of plant-pathogen interaction mediated by small molecules during the invasion of *Fusarium graminearum*, Jia et al. (2019) found that the fungal infection or the use of exogenous fusaoctaxin A inhibited the expression of three CAS-like genes and chloroplast genes. Thus, the lowered susceptibility of wheat to *Fusarium* A, and the inhibition of photosynthesis provided some evidence that chloroplasts might play a key role in regulating early immune responses (de Torres Zabala et al., 2015). *Sclerotinia sclerotiorum* is a well-known necrotizing fungus that attacks many crops (Bolton et al., 2006). The CAS is involved in the defense response of *Arabidopsis thaliana* to *S. sclerotiorum*, and actively regulates the accumulation of salicylic acid (SA) by promoting the expression of SA biosynthesis-related genes, thereby enhancing the resistance of plants to *S. sclerotiorum* (Tang et al., 2020). HopAU1 is an immune inducer existing in *Pseudomonas syringae pv. actinidiae* (Psa) which can interact with *NbCAS* in tobacco. Thus, silencing the *NbCAS* by RNAi in *N. benthamiana* greatly attenuated HopAU1-triggered cell death, suggesting that HopAU1 targets CAS and enhances plant immunity. Further study showed that the overexpression of *NbCaS* in *N. benthamiana* significantly improved plant resistance against *Sclerotinia sclerotiorum* and *Phytophthora capsici* (Zhang et al., 2022). Taken together, the CAS also serves as a promising resistant-related gene for breeding new disease-resistant varieties.

## Future outlook

The CAS has an important role in facilitating Ca^2+^-mediated stomatal closure and is associated with several signaling molecules. As a specific protein located on the chloroplast thylakoid membrane, the CAS functions as the first messenger along the signal transduction pathway in plants. There is some evidence that CAS may participate in various photosynthetic processes such as CO_2_ fixation and protein phosphorylation. With further research, we could gain insights into the Ca^2+^-induced signaling pathway and the functional characteristics of CAS in plant cells. In the future, it is important to further explore the following: (i) the various relationships between CAS and the other calcium-linked signals; (ii) the potential cross-talk between CAS and light signaling at the chloroplast thylakoid membranes; (iii) the CAS and its specific role in facilitating plant abiotic and biotic stress responses using advanced techniques/tools (proteomics, transcriptomics, and bioinformatics, etc.); (iv) extrapolating the understanding of CAS in model plants to non-model plants such as the legumes and horticultural crops, etc.

## Author contributions

YL, RB, CB, XH, and JY are responsible for the general review described in the manuscript. YL, XH, and JY provided further discussion and edited the manuscript. All authors reviewed and approved the final version of the submitted manuscript.

## Funding

This research was funded by the National Natural Science Foundation of China (project nos. 31772391 and 31301842), ARC-Linkage Project (project no. LP200100341), and the Scientific Research Project of the Education Department of Liaoning Province, China (project no. LJKZ0661).

## Acknowledgments

We sincerely thank the reviewers for all their valuable comments and recommendations to improve the manuscript.

## Conflict of interest

The authors declare that the research was conducted in the absence of any commercial or financial relationships that could be construed as a potential conflict of interest.

## Publisher’s note

All claims expressed in this article are solely those of the authors and do not necessarily represent those of their affiliated organizations, or those of the publisher, the editors and the reviewers. Any product that may be evaluated in this article, or claim that may be made by its manufacturer, is not guaranteed or endorsed by the publisher.
